# An ode to the 16S rRNA gene: its history, importance, caveats and future in microbiome research

**DOI:** 10.1099/mgen.0.001823

**Published:** 2026-08-25

**Authors:** F.J. Whelan, L.J. Hall

**Affiliations:** 1 School of Biological Sciences, Faculty of Biology, Medicine and Health, University of Manchester, Manchester, UK; 2 Department of Microbes, Infection and Microbiomes, School of Infection, Inflammatory and Immunology, College of Medicine and Health, University of Birmingham, Birmingham, UK; 3 Food, Microbiome and Health, Quadram Institute Bioscience, Norwich Research Park, Norwich, UK

**Keywords:** 16S rRNA gene sequencing, microbiome research

## Abstract

The 16S rRNA gene has - and continues to - play an important role in microbiology. It’s universality across prokaryotes and variation across species has allowed the sequencing of its hypervariable regions to be used to distinguish taxa within complex mixed bacterial communities. Although 16S rRNA gene sequencing has transformed our understanding of microbial communities, its use comes with important caveats and considerations, especially in light of the availability of whole-genome metagenomic sequencing. Within, we discuss the 16S rRNA gene and its important role in microbiome research, in the past, present, and future.

**Impact Statement**

The 16S rRNA gene has had a huge impact on the progress of microbiology and microbiome research. As new technologies such as whole-genome metagenomic sequencing become available, it is important for the community to consider the placement of 16S rRNA gene sequencing, its importance, and when it is most appropriate technology to use.

## Data Summary

**Fig. 1.**
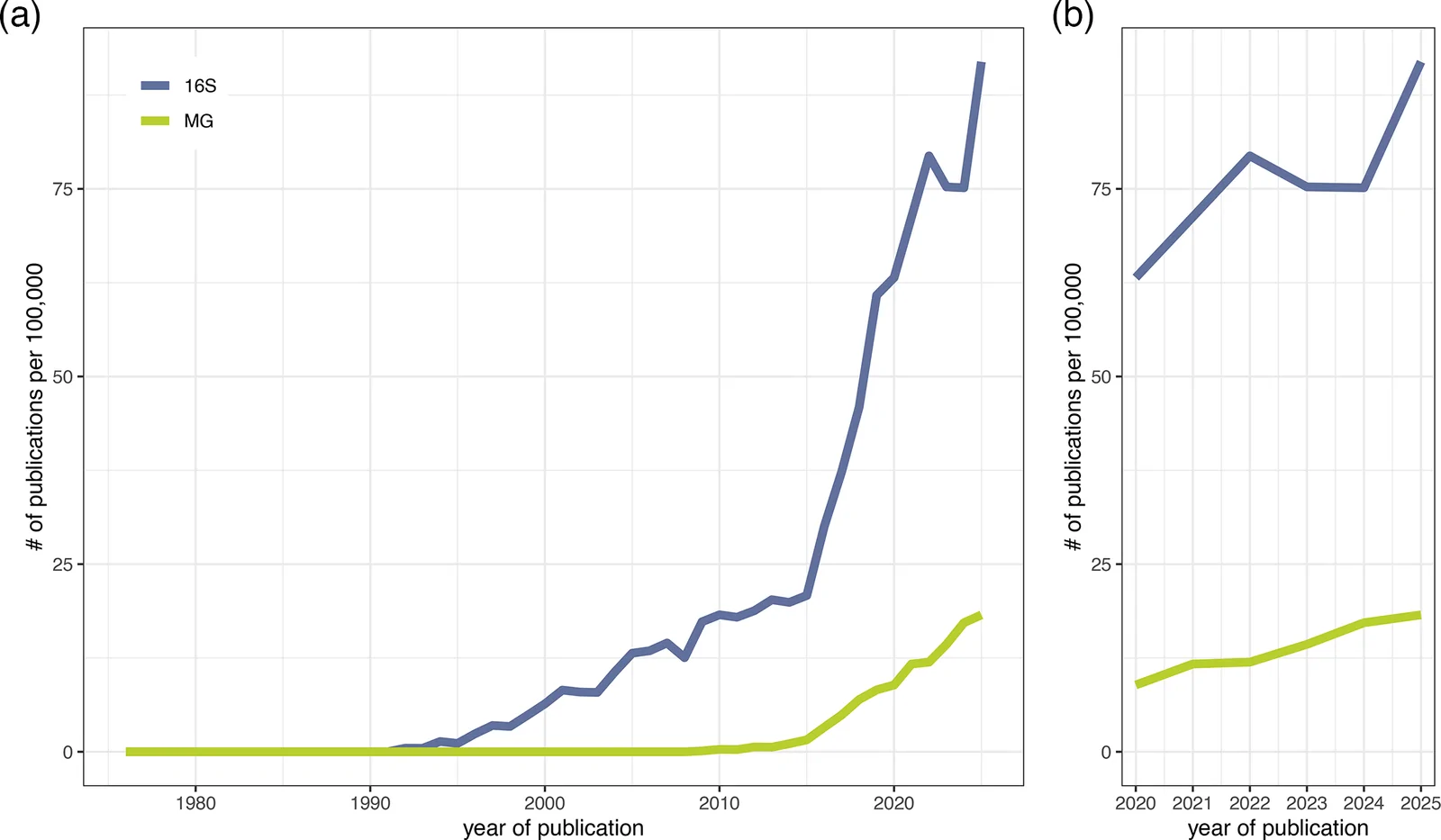
Occurrences of the terms ‘16S rRNA gene sequencing’ and ‘shotgun metagenomics’ in peer-reviewed manuscripts. Data were obtained from PubMed by year (https://github.com/esperr/pubmed-by-year) and plotted using R [52]. The data is based on the occurrence of each term in the full text of the paper since 1976 (**a**) and in the last 5 years (**b**). Data obtained 16 July 2025.

No new data were generated in this work. The data underlying Fig. 1 is publically available via PubMed and was plotted using open source software (https://github.com/esperr/pubmed-by-year).

## Origins and use: what 16S rRNA gene sequencing gets right

 The past two decades have seen an explosion of research on microbial communities, in large part due to the widespread adoption of 16S rRNA gene sequencing. The use of the 16S rRNA gene for bacterial taxonomic identification and definition of phylogenetic relationships was pioneered in the 1970s by Carl Woese *et al*. [1, 2] and was advanced by Norman Pace and colleagues in the 1980s [3, 4] with the application to mixed microbial communities. Early studies involved labourious generation of clone libraries and subsequent Sanger sequencing [5]; however, uptake accelerated with next- (or second-) generation platforms, including 454 pyrosequencing [6] and Illumina paired-end reads [7] and now includes long-read sequencing [8].

The 16S rRNA gene was chosen because of its universality across prokaryotes, its high sequence conservation and the infrequency of its transfer horizontally between species [2, 3]. Furthermore, 16S rRNA’s looped RNA structure of highly conserved stems and hypervariable loops meant that (almost) universal primers could be designed to the conserved gene regions to amplify the hypervariable loops. Generally, the sequences of the hypervariable regions of the 16S rRNA gene are unique to a given species or genus [9]; however, even full-length 16S rRNA gene sequences cannot always resolve taxa (e.g. differentiating *Shigella* species and *Escherichia coli*) [10, 11].

Platforms, such as Illumina’s MiSeq originally offered 150 bp paired-end reads, long enough to sequence some of the shorter variable regions (e.g. variable region 3, v3) or to obtain partial coverage of longer regions (e.g. v3–5) of the gene. The targeted variable region(s) differ across studies and large projects, including v1–3 and v3–5 in the Human Microbiome Project [12, 13], v4 in the Earth Microbiome Project [14] and v1–3 in the Microbial Database for Activated Sludge project [15]. Innovative techniques to ‘barcode’ the 16S rRNA gene amplicons from a given microbial community by the incorporation of unique 6-bp barcodes in the primer design [7] meant that many microbiome samples could be multiplexed onto a single Illumina sequencing run and, later, the reads separated by barcode.

The widespread adoption of high-throughput 16S rRNA gene sequencing has greatly enhanced our understanding of microbial communities. The impact has been felt in many diverse fields: 16S rRNA gene sequencing has predicted the ability of soil microbiomes to respond to climate change [16], indicated a ‘core microbiome’ of temperate trees [17] and a better understanding of ‘culture-negative’ bovine mastitis cases [18]. However, the place where 16S rRNA gene sequencing has perhaps been used most is in the study of the human microbiome. In this context, 16S rRNA gene amplicon sequencing has identified the influence of birth mode on the newborn microbiome [19], effects of antibiotics on the gut microbiome [20], the differences in microbial composition worldwide [21] and the identification of microbial communities in sites previously assumed sterile, such as the human lung [22, 23]. Community-level differences between health and disease have been commonly identified for gut diseases/disorders, such as inflammatory bowel disease (IBD) [24] and irritable bowel syndrome [25]. Additional studies have pinpointed particular changes in the relative abundance of key microbes and their correlation with human, animal and environmental health; for example, *Faecalibacterium prausnitzii* abundance is often reduced in individuals with various gut disorders [26]. None of these studies would have been possible without important innovations in bioinformatic and statistical analysis methods, including but certainly not limited to mothur [27], QIIME [28], DADA2 [29] and phyloseq [30].

## Critical caveats (and how to address them)

However, like all good things, 16S rRNA gene sequencing has important caveats that must be considered and accounted for. First, many microbial communities contain mixes of bacteria, archaea, fungi, viruses and other eukaryotic species, yet 16S rRNA gene sequencing is only able to capture the bacterial and archaeal diversity within these. Other amplicons – such as those targeting the 18S rRNA gene or internal transcribed spacer (ITS)1 and ITS2 – can help to elucidate some of these other members but others, such as bacteriophages and eukaryotic viruses, lack a universally conserved DNA target for amplicon sequencing. Additionally, 16S rRNA gene sequencing is inherently relative and cannot provide absolute counts. Community profiles can only indicate the proportional divisions of each taxon in the sample and are sensitive to microbial load. It can also only tell which bacteria are present but not their complement of genes – and thus, functionality. Furthermore, the ‘universal’ 16S rRNA gene primers developed for various hypervariable regions are not always quite as universal as hoped. For example, commonly used primers to amplify the v1–3 regions of the 16S rRNA gene have been shown to miss key health-associated genera, such as *Bifidobacterium* [31]. Further challenges in accurately interpreting the relative abundance of a species arise from differences in 16S rRNA gene sequence copy number (and, sometimes, the existence of distinct 16S rRNA genes within the same genome) [32, 33].

A critical caveat to consider when designing 16S rRNA gene sequencing studies is the potential for contamination [34]. Because 16S rRNA gene sequencing relies on amplification using primers universal to all prokaryotes, the method is prone to contamination. This is especially true of low-biomass samples where even low-level contamination can largely influence the resultant sequencing. Contamination can occur at multiple points from sample to sequence. First, contamination can be introduced during DNA extraction due to contaminants in commercial extraction kits and laboratory reagents [35]; in a seminal paper from 2014, the authors extracted DNA from a pure culture of *Salmonella bongori* at three different institutes, identifying at least 21 contaminating genera, the majority of which were consistent across laboratories, suggesting their presence in commercial reagents [35]. Most of these bacteria – for example *Hoeflea spp*. – are less commonly observed bacteria that wouldn’t be expected to be found in many niches (e.g. human gut) for which microbiome studies are conducted; however, others – for example, *Escherichia spp*. – are common members of many different environments and thus harder to distinguish from contaminant backgrounds [35]. Importantly, the relative abundance of contaminants observed increased as the amount of input *S. bongori* cells decreased, indicating the additional importance of mitigation against contamination when working with low-biomass samples [35]. Colloquially referred to as the ‘kitome’, the authors make recommendations on how to reduce the impact of contamination on low-biomass samples, which were later further outlined in opinion pieces [36] and consensus statements [37]. These recommendations include sequencing (a) sampling negatives (i.e. to control for contamination at the sampling source); (b) DNA extraction negatives (i.e. DNA extraction with no input sample) and positives (i.e. inclusion of a mock or synthetic community of known composition where contamination would be easy to identify); (c) PCR negatives (i.e. DNA extraction reagents) and positives (i.e. isolated DNA from a mock/synthetic community) [37]. Additionally, contamination can be mitigated by randomizing the extraction and sequencing of samples across treatment groups and/or other metadata variables and recording which samples were processed with which reagents and kits [35, 37]. Calls-to-action are being made to control for contamination in microbiome studies from environmental [37], clinical [38], animal [39] and insect [40] sources.

Despite best practices, contamination is still a real possibility. To help combat this are multiple *in silico* softwares which have been developed to aid in the removal of contaminant species. For example, Decontam is a statistical R package which identifies contamination based on increased abundance in low-concentration samples and presence in negative controls [41]. microDecon compares the proportion of potential contaminants in negative controls to sample datasets [42]. SourceTracker uses a Bayesian approach to determine potential contaminants and their source environments [43].

## Metagenomics complements – not replaces – amplicon surveys

Despite these caveats, 16S rRNA gene sequencing has been used in thousands of peer-reviewed publications since the 1970s (Fig. 1a). However, in recent years, the use of 16S rRNA gene sequencing has been plateauing alongside an increase in the use of whole-genome metagenomic sequencing (Fig. 1a and 1b). Metagenomics – the sequencing of the totality of the DNA present in a given sample [44] – is the natural next step from amplicon sequencing and addresses some of the caveats of 16S rRNA gene amplicons. Originally defined in 1998 [44] and used in 2002 [45], metagenomics is not limited to identifying only bacterial DNA but sequences the DNA of all organisms present in a sample. For communities, such as the human gut microbiome with its known diversity of fungi, viruses and free-living phage [46], metagenomics has allowed further characterization of these organisms as part of these mixed microbial communities. Metagenomics also allows better functional inference of the community by capturing the genomic content of at least the highly abundant organisms (dependent on the sequencing depth used and amount of non-microbial DNA present). Metagenomics does, however, still suffer from similar issues of contamination in low-biomass samples and community composition still being reported as ratios and not absolute counts.

Despite the wide-scale recent adoption of metagenomic sequencing, we argue that 16S rRNA gene sequencing is still a useful methodology and should be considered the technology of choice depending on the community being studied and the scientific question being asked. First, 16S rRNA gene amplicons are a relatively inexpensive way of identifying who is present in a community and how that presence might change over time, individual, disease state, etc. Its low cost means that sample sets containing thousands of samples can be assessed where similar numbers of shotgun metagenomics might be beyond the research budget or computational/bioinformatic capacity. It is unhelpful, for example, as a reviewer to suggest that metagenomics must be used if the research question can be addressed with a cheaper, well-established technology. Second, the technology continues to improve, including improvements to long-read 16S rRNA gene sequencing which allows for full-length sequencing of the gene, making this technology powerful for genus- (and sometimes species-) level resolution [8]. Third, metagenomics may have limited use in communities with large amounts of human/host DNA. One of the advantages of metagenomics – its representation of the DNA from all organisms in a community – can also be its disadvantage in communities where the vast majority of that DNA is non-microbial. For example, metagenomic studies of the cystic fibrosis human lung microbiomes can result in up to 99% of human DNA in their raw sequencing [47–50]. Not only does this introduce ethical concerns [51] but it also means that 99% of the output of an already relatively more expensive technique must then be discarded.

## Conclusions

16S rRNA gene sequencing was first used by Woese to identify a new kingdom of life before going on to aid in the discovery of the role of the microbiome in gut diseases, such as IBD and Crohn’s. It has uncovered the unimaginable diversity of assorted environments and continues to be a useful technology for specific, focused research questions. We don’t think this influential gene – when used appropriately – has quite finished helping us reveal the magical world of microbial communities just yet.

## Abbreviations

IBD: inflammatory bowel disease
ITS: internal transcribed spacer
